# A multifunctional molecular ferroelectric with chiral features, a high Curie temperature, large spontaneous polarization and photoluminescence: (C_9_H_14_N)_2_CdBr_4_†

**DOI:** 10.1039/d1sc03964d

**Published:** 2021-09-07

**Authors:** Yu-Kong Li, Yuan-Yuan Lai, Ting-Ting Ying, Ding-Chong Han, Yu-Hui Tan, Yun-Zhi Tang, Peng-kang Du, Hao Zhang

**Affiliations:** Faculty of Materials Metallurgy and Chemistry, Jiangxi University of Science and Technology Ganzhou 341000 Jiangxi Province P. R. China tyxcn@163.com tangyunzhi75@163.com

## Abstract

Low-dimensional chiral organic–inorganic hybrid metal halides have attracted a lot of attention in recent years due to their unique intrinsic properties, including having potential applications in optoelectronic and spintronic devices. However, low-dimensional chiral molecular ferroelectrics are very rare. In this paper, we report a novel zero-dimensional molecular ferroelectric (C_9_H_14_N)_2_CdBr_4_ (C_9_H_14_N^+^ = protonated 3-phenylpropylamine), which has obvious dielectric and thermal anomalies and shows a high Curie temperature at 395 K. It crystallizes in the *P*2_1_ space group at room temperature, showing a strong CD signal, large spontaneous polarization (*P*_s_ = 13.5 μC cm^−2^), and a clear ferroelectric domain. In addition, it also exhibits a flexible SHG response. The photoluminescence spectrum shows that **1** has broadband luminescence. At the same time, compound **1** has a wide band gap, which is mainly contributed to by the inorganic CdBr_4_ tetrahedron. The high tunability of low-dimensional chiral molecular ferroelectrics also opens up a way to explore multifunctional chiral materials.

## Abstract 2

A novel molecular ferroelectric (C_9_H_14_N)_2_CdBr_4_ with high Curie temperature (*T*_c_ = 395 K), strong CD signal, large spontaneous polarization, clear ferroelectric domain and fluorescence characteristics.

## Graphical abstract

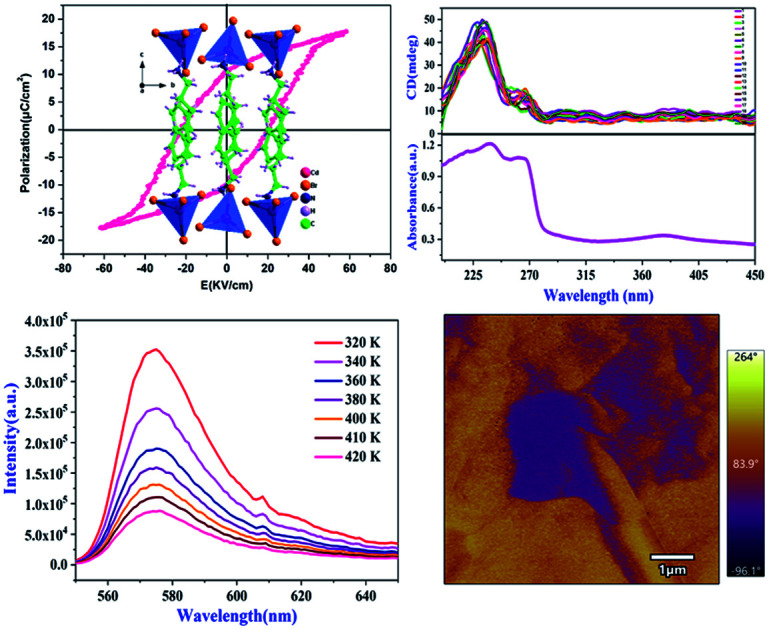

## Introduction

A ferroelectric is a kind of material which can realize spontaneous polarization and conversion with the action of an external electric field.^1^ It has wide application prospects in the field of intelligent materials and devices. Before the discovery of organic–inorganic hybrid metal halide type ferroelectrics, inorganic ferroelectric ceramics had been in the forefront of research for a long time because of their excellent piezoelectric properties.^2,3^ However, ferroelectric ceramics have little mechanical flexibility. Compared with traditional inorganic ceramic materials, organic–inorganic hybrid metal halide type multifunctional molecular ferroelectric materials have attracted great interest in recent years due to their high mechanical flexibility, environmental friendliness, and simple processing.^4,5^ These hybrid metal halides are the best candidates for ferroelectric phase transition materials because of their high structural adjustability, environmental protection and easy processing. At the same time, these materials show a wealth of photoelectric properties, including the ability to detect X-rays, luminescence and a tunable band gap.^6,7^ On the one hand, an organic–inorganic hybrid metal halide not only inherits the advantages of the organic part but also inherits the advantages of the inorganic part, which gives it the properties of a dielectric switch and photoluminescence in a compound. On the other hand, stable luminescent properties can be obtained by introducing metal ions into inorganic components.^8–10^ Although great progress has been made in research into ferroelectrics in the last ten years, the combination of ferroelectric materials and practical applications makes high-temperature phase transition materials more ideal,^11^ such as DIPAX (DIPA = diisopropylaminium; X = Cl, Br, ClO_4_^−^) (*T*_c_ = 426 K);^12^ hexane-1,6-diammonium pentaiodobismuth (HDA-BiI_5_) (*T*_c_ = 380 K) which has the highest phase transition temperature and the narrowest band gap (*E*_g_ = 1.89 eV);^13^ [(*R*)-*N*-(1-phenylethyl)ethane-1,2-diaminium]PbI_4_ (*T*_c_ = 378 K);^14^ and (4,4-difluoropiperidinium)_4_AgBiI_8_ (*T*_c_ = 422 K).^15^ Lead-free metal halides have been widely studied in photoelectric and photovoltaic fields. For example, Sn, Sb, Cd and Bi ions can be used to replace Pb ions, and can also show excellent photoluminescence and ferroelectric properties,^16–18^ such as [(C_3_H_5_)_2_N(CH_3_)_2_]_2_SnCl_6_ which shows excellent semiconductor and photoluminescence properties.^19^ However, Sn-based metal halides show low stability and it is easy to oxidize Sn^2+^ into Sn^4+^. Therefore, lead-free Bi-based and Cd-based hybrid metal halides are considered to be the most promising substitutes due to their low toxicity and high stability.^20^ There are only a few multifunctional organic–inorganic metal halide ferroelectrics containing the inorganic anion CdBr_4_^2−^ in the literature, such as (3-pyrrolinium)CdBr_3_ (*T*_c_ = 260 K),^21^ ([BrCH_2_CH_2_N(CH_3_)_3_]_2_^+^[CdBr_4_]^2−^) (*T*_c_ = 390 K),^22^ [cyclopentylammonium]_2_CdBr_4_ (*T*_c_ = 340 K),^23^ (diisopropylammonium)_2_[CdBr_4_] (*T*_c_ = 244 K),^24^ [BnNMe_2_R]CdBr_4_ (*T*_c_ = 400 K),^25^ and Cd-based molecular ferroelectrics with both chiral and luminescent properties are rare.

According to the theory of “ferroelectrochemistry”, by introducing chiral ligands into organic cations, organic–inorganic hybrid metal halides have unique chirality, which is conducive to the construction of chiral molecular ferroelectrics.^26^ Chirality is one of the basic properties of nature. According to the definition of crystallography, the ferroelectric phase necessarily crystallizes into 10 polar groups (*C*_1_, *C*_2_, *C*_S_, *C*_2v_, *C*_4_, *C*_4v_, *C*_3_, *C*_3v_, *C*_6_ and *C*_6v_), of which 5 are chiral groups (*C*_1_, *C*_2_, *C*_3_, *C*_4_, and *C*_6_).^27^ Ferroelectric Rochelle salt ([KNaC_4_H_4_O_6_]_3_·4H_2_O) was the first homochiral compound discovered in 1920.^28^ Although chiral molecular ferroelectrics are abundant, they have a high chance of crystallizing in chiral point groups. So far, recorded multifunctional chiral molecular ferroelectrics are still very rare.^29^

Based on the above experience, we report a zero-dimensional organic–inorganic hybrid ferroelectric (C_9_H_14_N)_2_CdBr_4_. Its Curie temperature *T*_c_ is 395 K. More interestingly, compound **1** crystallizes in the chiral space group *P*2_1_, showing a strong CD signal. At the same time, the clear ferroelectric domain reveals the ferroelectricity of compound **1**. Therefore, the discovery of compound **1** will help us to find multifunctional molecular ferroelectrics with a high Curie temperature.

## Results and discussion

### Differential scanning calorimetry (DSC) and the dielectric properties of compound **1**

**Fig. 1:**
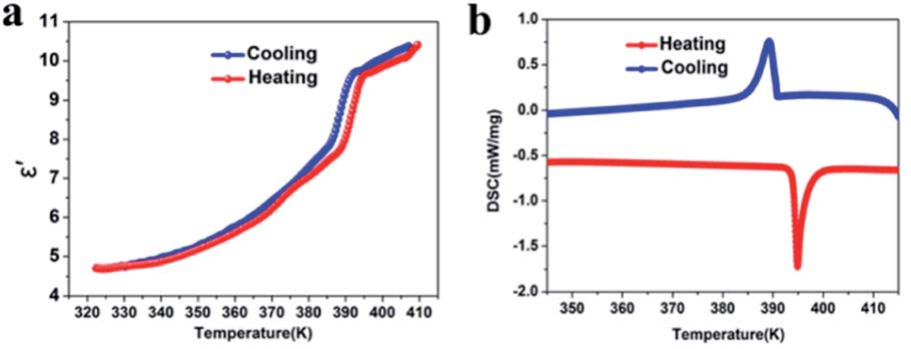
The temperature dependence of the dielectric constant (a) and DSC analysis (b) of compound **1**.

In order to confirm the reversible phase transition of compound **1**, DSC tests were carried out. The temperature range of 345–415 K (Fig. 1b) was selected. Under the heating/cooling cycle mode, the endothermic peak and exothermic peak of compound **1** were 395 and 389 K, respectively. In addition, a sharp reversible peak and large thermal hysteresis were observed in the DSC curves, which indicated that compound **1** belonged to a typical first-order phase transition.^30^ We further calculated that the entropy changes (Δ*S*) of compound **1** during heating and cooling are 15.95 J mol^−1^ K^−1^ and 15.15 J mol^−1^ K^−1^, respectively. According to the Boltzmann equation Δ*S* = *nR* ln(*N*) (*N* represents the possible orientation), the estimated *N* values are 6.75 and 6.17 (ESI†), respectively, significantly larger than 1, suggesting that the phase transition is an ordered–disordered type.^6^ In addition, since the melting point of compound **1** is 450 K, we chose a temperature range of 120–440 K for the DSC test (Fig. S7, ESI†). The results show that there is only a thermal anomaly peak of 395 K in this temperature range. At the same time, we used a scan rate of 15 K min^−1^ to test compound **1** for 7 cycles (Fig. S8, ESI†), and the results show that even after 7 endothermic and exothermic cycles, the phase transition can still maintain the initial endothermic and exothermic effect. This shows that compound **1** can be used as an excellent phase change energy storage material.^31^ It is worth noting that the transition temperature is higher than that of Cd-based molecular ferroelectrics reported previously, such as [C_6_H_5_(CH_2_)_4_NH_3_]_2_CdCl_4_ (*T*_c_ = 340 K),^32^ [cyclopentylammonium]_2_CdBr_4_ (*T*_c_ = 340 K),^23^ or (diisopropylammonium)_2_[CdBr_4_] (*T*_c_ = 244 K),^24^ and it is comparable to the phase transition temperature of TMCM-CdCl_3_ (*T*_c_ = 400 K),^33^ [BnNMe_2_R]CdBr_4_ (*T*_c_ = 400 K).^25^ In addition, we measured TG-DTA (Fig. S6, ESI†). The results show that compound **1** decomposes above 595 K and has good thermal stability, which is much higher than the structural phase transition temperature (*T*_c_ = 395 K).

In order to further confirm the structural phase transition of compound **1**, we measured its dielectric properties. Generally speaking, once the structural phase transition occurs, it will lead to an abnormal transition from a low dielectric state to a high dielectric state.^34^ Therefore, the measurement of dielectric properties is considered to be a reliable method to test the structural phase transition. We used compound **1** powder to make pressed tablets and measured the change in dielectric constant (frequency 1 MHz) during heating and cooling. As expected, compound **1** showed a significant step-like dielectric anomaly near the phase transition temperature (Fig. 1a). At the same time, we calculated its dielectric constant, during heating, at 320–395 K (low dielectric state), when the dielectric constant rises in the range of 5–7.5 (a.u.), but when the temperature is greater than 395 K (high dielectric state), the dielectric constant rises sharply to 9.5 (a.u.) and finally remains stable at about 10 (a.u.), which is consistent with the change in the DSC curve. The significant dielectric switching during the heating and cooling process makes compound **1** an important energy storage and switchable dielectric switching material.^35,36^

### Crystal structure of compound **1**

**Fig. 2:**
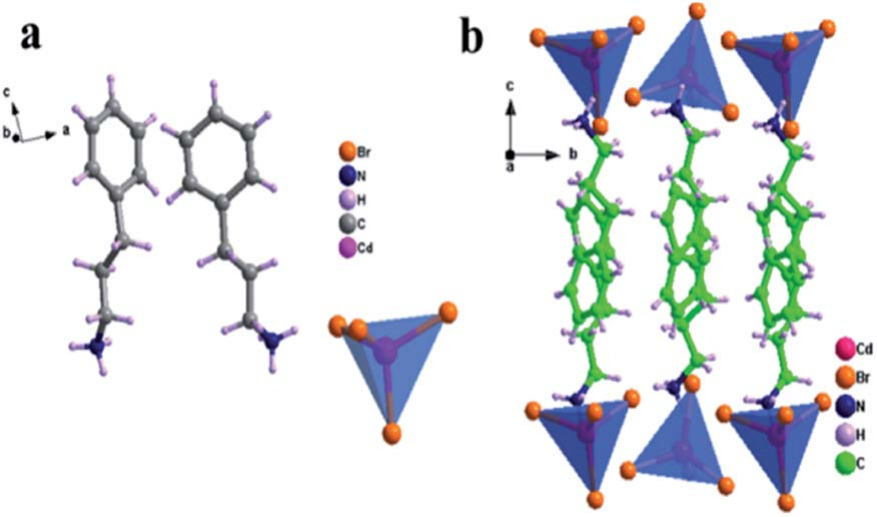
An asymmetric unit diagram of compound **1** (a). A packing view of compound **1** at 300 K viewed along the *a*-axis (b).

In order to understand the ferroelectric phase transition mechanism, we measured the single crystal structure of compound **1** at 300 K (LTP). Under LTP, compound **1** crystallizes in a monoclinic polar chiral ferroelectric space group *P*2_1_ (point group 2 with two symmetry elements (*E*, *C*_2_)) with cell parameters *a* = 11.1342(6) Å, *b* = 7.8950(4) Å, *c* = 13.9106(7) Å, *α* = *γ* = 90°, *β* = 96.953(2)°, *Z* = 2, and *V* = 1213.81(11) Å^3^. As shown in Fig. 2a, the asymmetric unit of compound **1** in LTP is composed of two (C_9_H_14_N)^+^ cations and a twisted [CdBr_4_]^2−^ anion tetrahedron, which satisfies the conservation of charge. The bond length and angle are shown in Tables S2 and S3† (ESI^†^), where the distance range of the Cd–Br bond is 2.5599(14) Å–2.5998(13) Å, and the angle range of Cd–Br–Cd is 104.81(5)°–113.96(5)°. Two ordered organic cations (C_9_H_14_N)^+^ are simultaneously distributed on one side of the [CdBr_4_]^2−^ anion tetrahedron to balance the charges, and are connected by *N*–H⋯Br hydrogen bonds to form an infinite *sigmoid* chain along the *b*-axis (Fig. S12, ESI†). The distance range of N⋯Br is 3.374(10) Å–3.556(9) Å, and the corresponding *N*–H⋯Br angle range is 114°–155°. The detailed hydrogen bond data is shown in Table S4 (ESI†). At the same time, it can be seen from the stacking diagram (Fig. 2b) that the organic cations are orderly embedded in the interlayer formed by the inorganic anion [CdBr_4_]^2−^ tetrahedron, and are connected together only by weak hydrogen bonding, which provides more freedom for the movement of organic cations, thus causing the occurrence of the ferroelectric phase transition.^37^ In addition, viewed from the *a*-axis, the NH_3_ group on the (C_9_H_14_N)^+^ cation is located in the gap between adjacent tetrahedrons, resulting in a polarization stack along the positive *b*-axis (Fig. S11, ESI†).

**Fig. 3:**
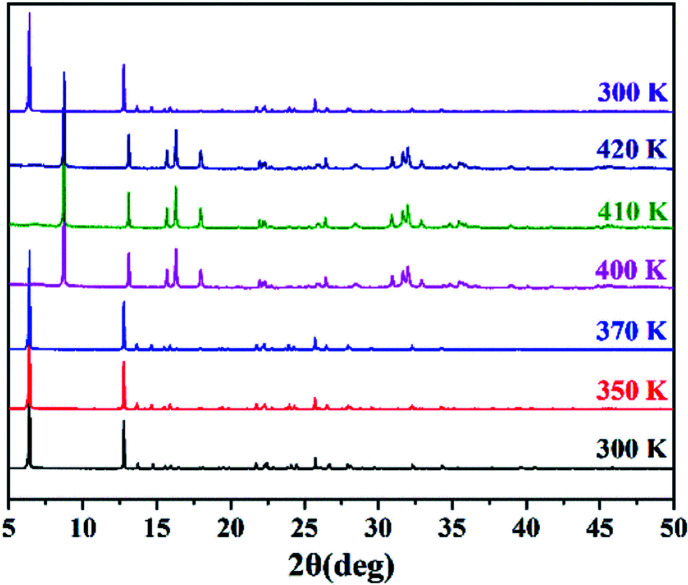
Variable-temperature PXRD patterns of **1**.

Due to the poor high-temperature single-crystal diffraction data for compound **1**, the complete crystal structure of compound **1** cannot be resolved. Fortunately, we confirmed the structural phase transition by variable temperature PXRD (Fig. 3). When heated from 300 K to 370 K, the diffraction peak of compound **1** did not change, indicating that the structure remained stable over a wide temperature range below *T*_c_. With an increase in temperature, the number of diffraction peaks in the PXRD pattern at 400–420 K changes significantly. In addition, when the temperature was lowered to 300 K again, the PXRD pattern of compound **1** returned to its original state, indicating that it had undergone a reversible phase transition. As expected, the temperature-variable PXRD results are consistent with the DSC results. At the same time, we carried out Le Bail refinements on the PXRD data in HTP to gain insight into some structural information (Fig. S5, ESI†). We infer that the unit cell parameters of compound **1** in the high temperature phase (HTP) are *a* = 7.958 Å, *b* = 8.008 Å, *c* = 20.222 Å, *α* = *β* = *γ* = 90°, and *V* = 1288.7 Å^3^. In view of the chirality and orthorhombic crystal system, the high temperature phase of compound **1** may adopt a 222-point group, among which the obtained most likely space group was determined to be *P*222 (see the ESI† for detailed refinement results). More specifically, this symmetry breaking occurs with an Aizu notation of 222F2.

### Chiral characterization

**Fig. 4:**
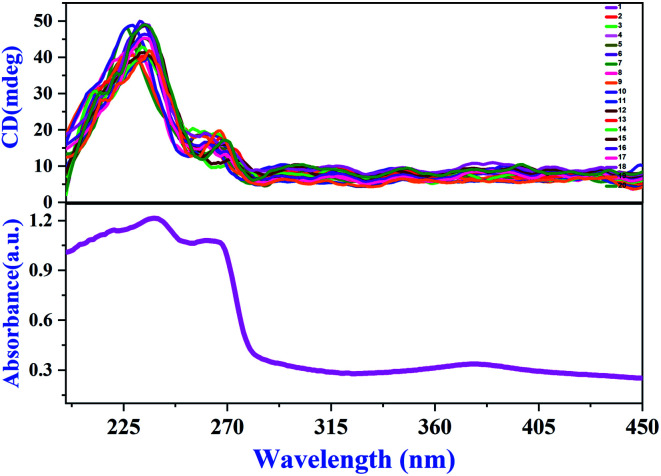
Solid-state CD spectra of **1** and the corresponding absorption spectrum.

At room temperature, compound **1** crystallizes in chiral and polar group *P*2_1_. In order to further verify the chiral feature of LTP single crystals, we randomly selected 20 complete single crystals and measured their solid-state CD spectra.^38^ As shown in Fig. 4, the peak pattern of 20 groups of CD spectra at *λ* = 235 nm is basically the same, corresponding to the absorption peak of the UV absorption spectrum, and the strong Cotton effect is positive. Therefore, through the solid-state CD spectrum results and Flack absolute structure parameters (Table S1, ESI†), we can determine that compound **1** is a ferroelectric with a single chiral feature. At present, there are relatively few ferroelectrics with a single chiral feature, so compound **1** will have great application prospects in medicine, chemistry and other fields.^39,40^

### SHG response and ferroelectric properties

**Fig. 5:**
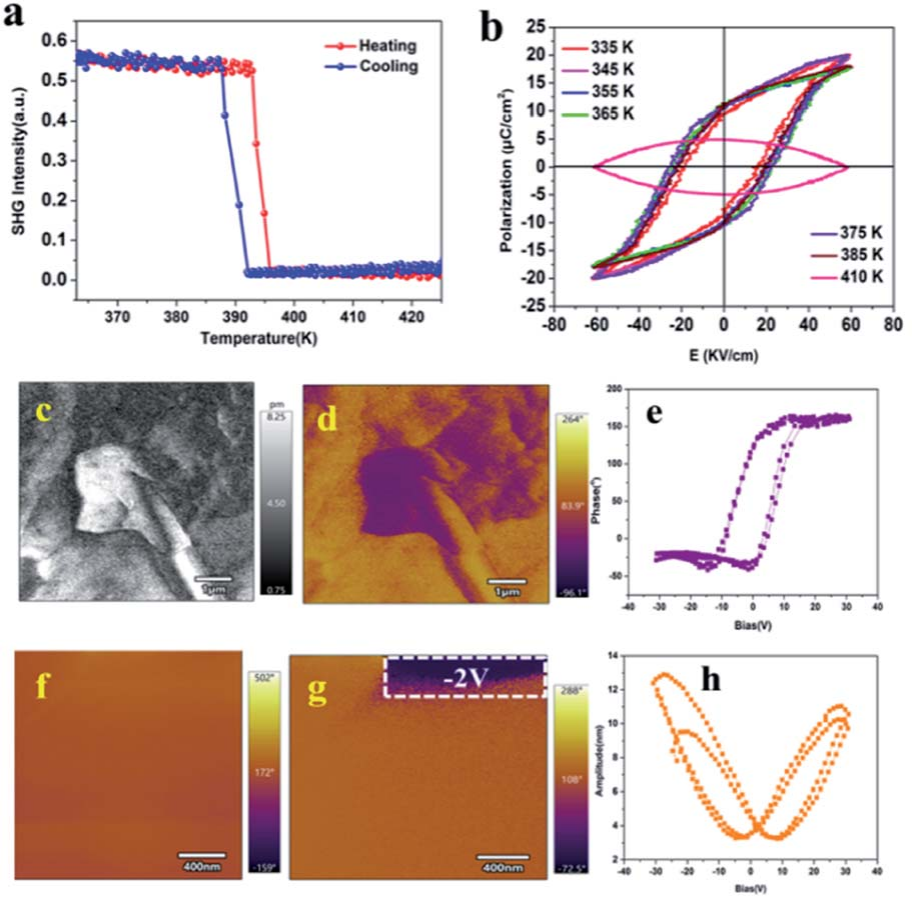
The variable-temperature SHG signal of **1** (a) and polarization hysteresis loops at different temperatures of compound **1** (b). The lateral PFM amplitude (c) and phase mapping (d). An amplitude–voltage butterfly loop (e) and phase–voltage hysteresis loop (h). Pristine PFM mapping for a region of 400 nm × 400 nm (f). A lateral PFM phase image after applying a DC bias voltage of −2 V in the white box (g).

Compound **1** crystallizes in the polar point *P*2_1_ space group. Therefore, we measured the change in its SHG signal intensity with temperature. The SHG signal is very sensitive to the transition from a centrally symmetrical phase to a non-centrally symmetrical phase, so the SHG measurement can also verify the occurrence of a structural phase transition.^41,42^ As shown in Fig. 5a, before the phase transition temperature (LTP), a clear SHG signal can be observed in compound (C_9_H_14_N)_2_CdBr_4_, and the intensity is stable at 0.55 (a.u.), indicating that **1** is noncentrosymmetric in LTP, because only the noncentrosymmetric space group SHG is active. As the temperature increases, the SHG intensity continues to remain at 0.55 (a.u.). When the temperature increases to about 395 K (HTP), the SHG signal intensity drops sharply to 0 (a.u.), which clearly indicates that compound **1** belongs to the centrosymmetric space group in HTP. At the same time, the change trend in SHG signal intensity conforms to the first-order phase transition characteristics.^43^

A polarization hysteresis loop (*P*–*E*) test is the main evidence to prove whether it has ferroelectricity.^44^ A *P*–*E* hysteresis loop with an ideal shape is obtained by using the Sawyer–Tower circuit method, which shows that it is a ferroelectric material. As shown in Fig. 5b, the ferroelectric properties of compound **1** were measured by measuring its hysteresis loops in the temperature range of 335–410 K. The *P*–*E* hysteresis loop shows a well-defined rectangle in the temperature range from 335 K to 385 K, and the saturated *P*_s_ values varied from 12.5 to 13.5 μC cm^−2^ (as highlighted in different colors), which confirms the existence of ferroelectricity in LTP, and the temperature change has little effect on spontaneous polarization before the phase transition temperature. When the temperature rises to 410 K (higher than *T*_c_), the shape of the *P*–*E* hysteresis loop becomes round rather than straight due to leakage current, which indicates that **1** loses ferroelectricity.^15^ At the same time, we tested the polarization hysteresis loop of 150–200 K, which also shows an ideal rectangle (Fig. S9, ESI†). It is worth noting that the spontaneous polarization *P*_s_ of compound **1** is much larger than those of reported zero-dimensional metal halide ferroelectrics, such as [C_6_H_5_CH_2_CH_2_NH_3_]_2_[CdI_4_] (0.36 μC cm^−2^),^34^ (diisopropylammonium)_2_[CdBr_4_] (0.39 μC cm^−2^),^24^ or [cyclopentylammonium]_2_CdBr_4_ (0.57 μC cm^−2^),^23^ and is comparable to [BnNMe_2_R]CdBr_4_ (Bn = benzyl; R = Me; R = *n*-Pr) (14.24 μC cm^−2^).^25^ For Cd-based halide molecular ferroelectrics, there are some one-dimensional ferroelectrics which also have excellent ferroelectric and piezoelectric properties, such as the 1D Cd-based molecular ferroelectric TMCM-CdCl_3_ reported by Xiong *et al.*^33^ It has a perfect ferroelectric hysteresis loop and a high Curie temperature. It is worth noting that its piezoelectric coefficient *d*_33_ = 1540 pC N^−1^ is comparable to that of high-performance ceramic materials. Our subsequent work will be devoted to exploring ferroelectrics with piezoelectric properties.

In order to further confirm the ferroelectric behavior of compound (C_9_H_14_N)_2_CdBr_4_, the piezoelectric response force microscopy (PFM) measurement of its thin film was carried out, which is the most powerful method to characterize ferroelectric materials.^45^ The ferroelectric domain is imaged by scanning the surface of the film in contact mode and applying an AC voltage through a conductive probe. The lateral PFM amplitude and phase images of (C_9_H_14_N)_2_CdBr_4_ single crystal thin films are shown in Fig. 5c and d, respectively. It can be seen that the phase image exhibits 180° contrast of domain orientation. The domain walls, where the signal is a minimum in the amplitude image, separate the adjacent domains.^43^ At the same time, we measured the vertical amplitude and phase diagram (Fig. S10, ESI†). The results show that there is no obvious ferroelectric domain, which indicates that the ferroelectrics have only one allowed polarization direction. It is confirmed that compound **1** belongs to uniaxial ferroelectrics. The typical hysteresis loops (Fig. 5e) and butterfly loop (Fig. 5h) further confirm that a thin film of compound **1** has good ferroelectric switching properties.^46^

The inherent characteristic of ferroelectrics is switchable spontaneous polarization.^15^ In order to verify the switchable spontaneous polarization of compound **1**, we first measured the local lateral PFM phase and amplitude on the surface of the single crystal sample thin film in the 400 nm × 400 nm region. As shown in Fig. 5f, the PFM signal is uniform and single.^2^ When a −2 V DC bias voltage is applied to the white frame, the polarization direction is reversed, and the color of the corresponding phase image changes from brown to purple (Fig. 5g). The measurement result of PFM proves that **1** has a switchable spontaneous polarization characteristic.

### Photoluminescence and UV-vis absorption

**Fig. 6:**
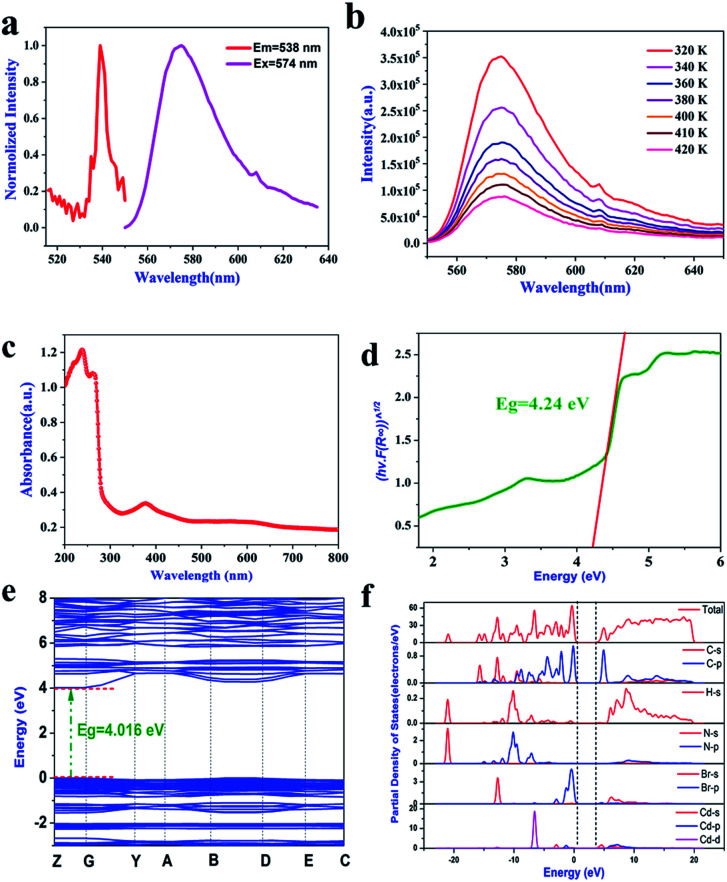
(a) Fluorescence spectra of compound **1** at room temperature. (b) Temperature-dependent PL spectra of crystal **1**. (c) The UV-vis absorption spectrum of compound **1**. (d) The calculated optical band gap of compound **1**. (e) The band structure of compound **1**. (f) Partial density of states of compound **1**.

We further evaluated the photoelectric properties of **1** by measuring the photoluminescence (PL) and UV-vis absorption spectra. As shown in Fig. 6a, compound **1** not only has switchable thermodynamic properties, but also interesting photoluminescence properties.^47^ At 538 nm excitation wavelength, a strong photoluminescence (PL) emission corresponding to yellow emission was observed at 574 nm. At the same time, in order to understand the effect of a structural phase transition on the photoluminescence characteristics, we also carried out temperature-dependent photoluminescence (PL) measurement, where the measurement temperature range was 320–420 K, as shown in Fig. 6b. No matter what the temperature, a strong emission peak can be observed at 574 nm, and its position does not change with temperature. Therefore, the structural phase transition (395 K) does not significantly affect the photoluminescence emission wavelength.

We measured the UV-vis absorption spectrum in the wavelength range of 200–800 nm. As shown in Fig. 6c, compound **1** has a sharp absorption edge at 255 nm, indicating that compound **1** has a direct band gap.^48^ Meanwhile, according to the fitting calculation of the Tauc equation (Fig. 6d), the direct band gap value of compound **1** is 4.24 eV. Compared with the organic–inorganic hybrid metal halide ferroelectric ([BrCH_2_CH_2_N(CH_3_)_3_]_2_^+^[CdBr_4_]^2−^) (*E*_g_ = 4.96 eV) containing the same inorganic anion [CdBr_4_]^2−^,^22^ the band gap value is smaller, and it is less than that of [C_3_H_5_NH_3_]_2_CdCl_4_ (*E*_g_ = 5.20 eV).^49^ In order to further understand the semiconductor properties of compound **1**, we calculated its band structure and PDOS. As shown in Fig. 6e, the maximum value of the valence band and the minimum value of the conduction band are located in the same position of the Brillouin zone, and the band gap value is 4.016 eV, which is smaller than the experimental value, mainly due to defects in the DFT calculation. At the same time, according to the PDOS diagram (Fig. 6f), the minimum valence band of compound **1** is mainly controlled by the Br-4p orbital, and the maximum conduction band is controlled by the Cd-5s orbital. In conclusion, the band gap value of compound **1** is mainly contributed by the inorganic CdBr_4_ tetrahedron.

## Conclusions

In summary, we successfully synthesized a novel low-dimensional molecular ferroelectric (C_9_H_14_N)_2_CdBr_4_ (C_9_H_14_N^+^ = protonated 3-phenylpropylamine) which showed a Curie temperature as high as 395 K and spontaneous polarization of 13.5 μC cm^−1^.^2^ In addition, it also has a flexible SHG response and a strong CD signal. The photoluminescence spectrum shows that **1** has broadband luminescence. This discovery opens up a new way to explore new low-dimensional chiral molecular ferroelectrics, especially ferroelectric semiconductors with a high phase transition temperature and excellent photoelectric properties.

## Experimental

### Synthesis of compound **1**

All reagents and solvents are of commercial quality. 2 mmol of 3-phenylpropylamine and 1 mmol of CdBr_2_·4H_2_O were added to an aqueous solution (10 mL) containing hydro bromic acid (40 wt%). After stirring and dissolving at room temperature, a colorless transparent crystal was obtained (Fig S1, ESI†). And the crystal was not oxidized and did not absorb moisture when placed in the air for a long time. The phase purity of the crystal was confirmed by infrared spectroscopy (Fig. S2, ESI†) and powder X-ray diffraction (Fig. S3, ESI†).

### Thin-film preparation

The precursor solution of (C_9_H_14_N)_2_CdBr_4_ was prepared by dissolving 20 mg of the crystals in 500 μL of aqueous solution. Then, 20 μL of precursor solution was spread on a clean indium-doped tin oxide (ITO) glass substrate. The thin film of (C_9_H_14_N)_2_CdBr_4_ was obtained after annealing at 400 K for 30 min.

### Instrumentation

Powder X-ray diffraction (PXRD) patterns were carried out on a Rigaku D/MAX 2000 PC X-ray diffraction instrument with Cu radiation (*K*_α1_ = 1.54060 Å, *K*_α2_ = 1.54443 Å). The data were collected during the heating process in the temperature range of 300–420 K for *θ* in the range of 5–50°. DSC measurements were performed by heating/cooling the powder sample at a rate of 15 K min^−1^ on a PerkinElmer Diamond DSC instrument. Thermogravimetric analysis (TGA) measurement was performed on a TA-Instruments STD2960 system from room temperature to 1050 K at a rate of 10 K min^−1^ under a nitrogen atmosphere. The dielectric constant of compound **1** was measured with an Agilent or TH2828A impedance analyzer. During heating and cooling, the powder particle sample was measured at a rate of 5 K min^−1^. The SHG signals were measured throughout with an Edinburgh Instruments FLS 920 using a laser with low divergence (Nd: YAG, 1064 nm, 5 ns, 1.6 MW peak power, 10 Hz repetition rate). The laser was a Vibrant 355 II, OPOTEK. The ferroelectric hysteresis loop was measured on a standard RT 6000 ferroelectric tester (Albuquerque, USA). PFM measurements were performed by using PFM mode on an Asylum MFP-3D Infinity atomic force microscope. PL emission spectra were measured at room temperature on a spectra fluorophotometer (JASCO, FP-6500). UV-vis absorption spectra were obtained using a Shimadzu (Tokyo, Japan) UV-2550 spectrophotometer in the range of 200–800 nm. The solid-state CD spectrum for compound **1** was recorded on a Jasco-1500 CD spectropolarimeter at 300 K and for the wavelength range 200–450 nm.

### X-ray diffraction characterization

The single-crystal X-ray diffraction studies were performed with a Bruker Smart Apex II single-crystal diffractometer operating with a graphite-monochromated Mo-sealed tube source (*K*_α_ radiation, *λ* = 0.71073 Å). The crystal structure of **1** was determined at 300 K. The structures were solved and the models were refined using the SHELXS and SHELXL programs. The data collection and structure refinement of these crystals are summarized in ESI, Table S1.† The crystallographic information on the crystal structures of **1** determined at 300 K has been deposited in CIF format in the Cambridge Crystallographic Database Centre, CCDC: 2097252.

## Author contributions

Yun-Zhi Tang, Yu-Hui Tan, and Yu-Kong Li conceived the experiments and co-wrote the manuscript. Yu-Kong Li, Yuan-Yuan Lai, and Ting-Ting Ying performed the experiments. All the authors discussed the results.

## Conflicts of interest

There are no conflicts to declare.

## Supplementary Material

SC-012-D1SC03964D-s001

SC-012-D1SC03964D-s002
