# Supplementary material for: A multifunctional molecular ferroelectric with chiral features, a high Curie temperature, large spontaneous polarization and photoluminescence: (C9H14N)2CdBr4
Source: Chem Sci. 2021 Sep 7;12(39):13061–7. doi: 10.1039/d1sc03964d (PMC8513930; doi:10.1039/d1sc03964d)
Supplement: SC-012-D1SC03964D-s001 [file SC-012-D1SC03964D-s001.pdf]

Electronic Supplementary Material (ESI) for Chemical Science.
This journal is © The Royal Society of Chemistry 2021

Electronic Supplementary Information

Multifunctional molecular ferroelectric with Chiral feature, High Curie Temperature, Large Spontaneous Polarization and Photoluminescence: $(C_9H_{14}N)_2CdBr_4$

Yu-Kong Li, Yuan-Yuan Lai, Ting-Ting Ying, Ding-Chong Han, Yu-Hui Tan,* Yun-Zhi Tang,* Peng-kang Du, Hao Zhang

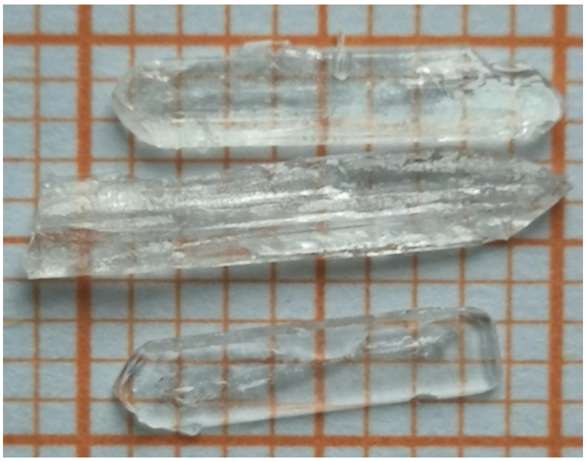

Fig. S1 Crystal morphology of compound **1.**

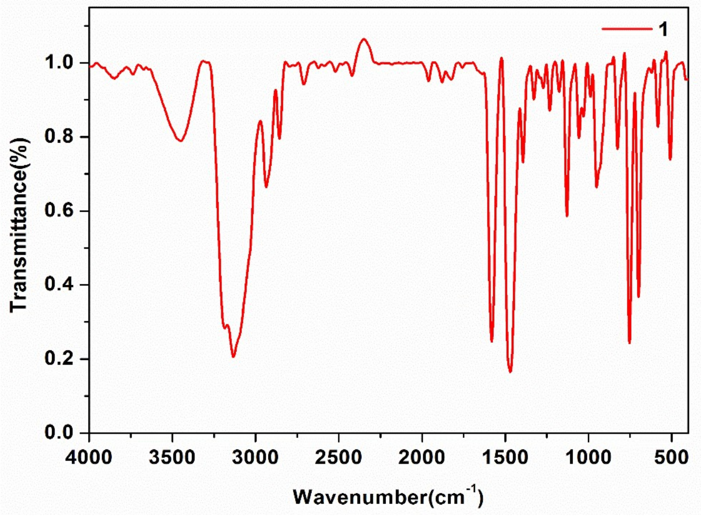

Fig. S2 Infrared spectrum of compound **1**.

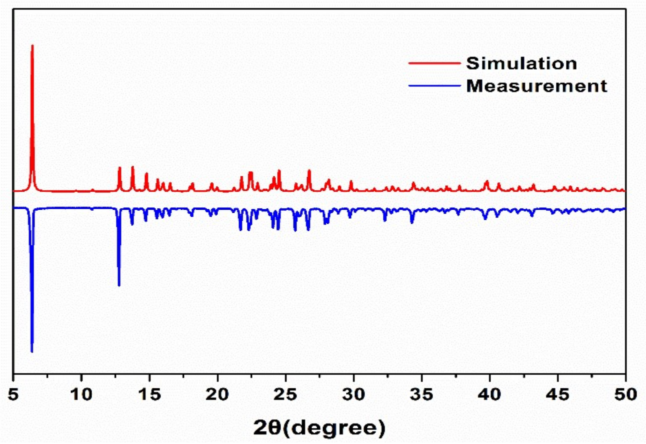

Fig. S3 The powder XRD of **1**.

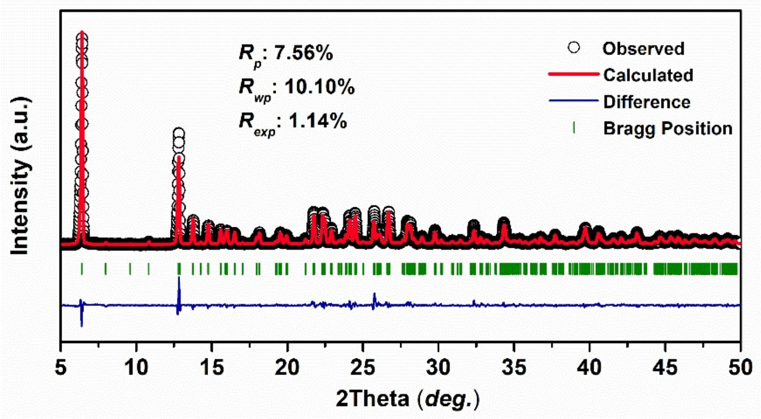

Fig. S4 Powder X-ray diffractograms of **1** collected in 300 K (**LTP**), refined by Le Bail method using the FULLPROF program. The lattice parameters obtained from the fitting: *a*= 11.15813(0.0046), *b*= 7.91511(0.00034), *c*= 13.94007(0.00061) Å (R_p = 7.56%, R_{wp} = 10.10%, R_{exp} = 1.14%).

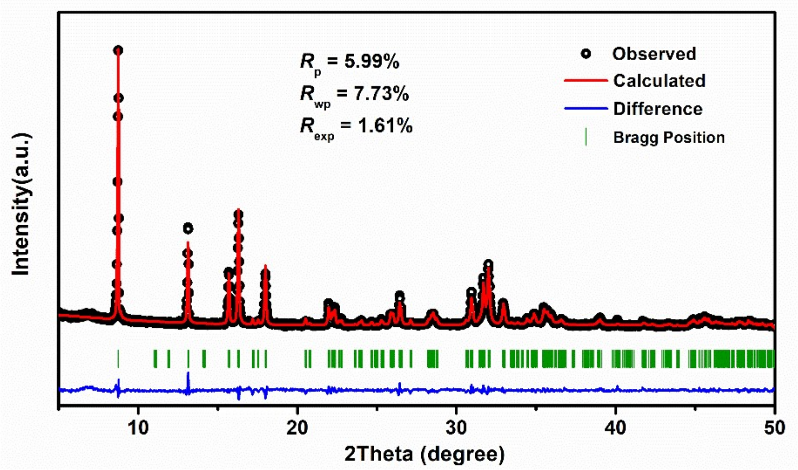

Fig. S5 Structural refinement results of PXRD data for compound **1** in 405 K (**HTP**). The indexing of PXRD data reveals an orthorhombic lattice, and through the Le Bail refinements, we obtained the orthorhombic point group mmm. The refined cell parameters are *a* = 7.958 Å, *b* = 8.008 Å, *c* = 20.222 Å, *α* = *β* = *γ* = 90°, *and V* = 1288.7 Å^3 (R_p = 5.99%, R_{wp} = 7.73%, R_{exp} = 1.61%). These results are highly consistent, confirming the phase purity of the compound and high accuracy of the simulation methods.

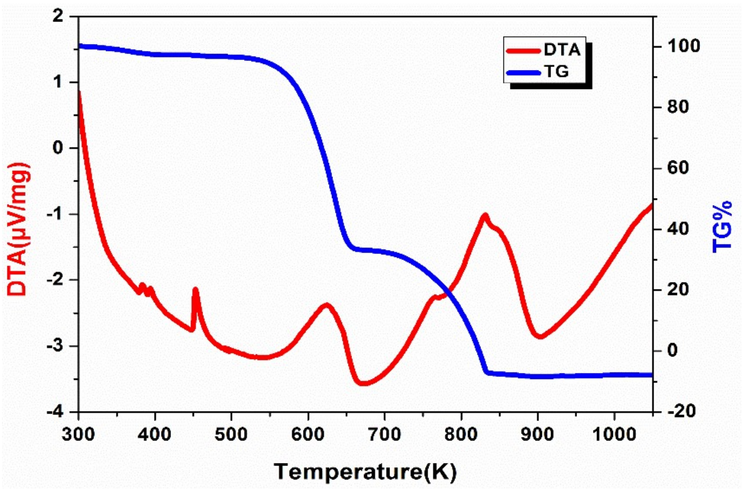

Fig. S6 TG-DTA curves for **1.**

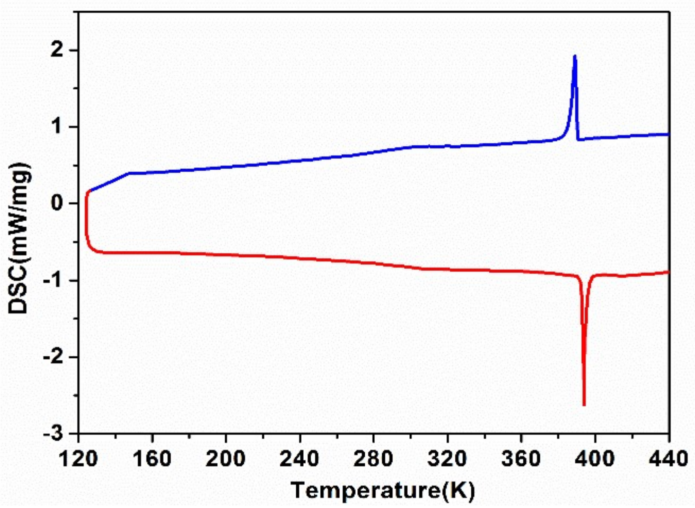

Fig. S7. DSC curve of compound **1**, temperature range 120-440 K.

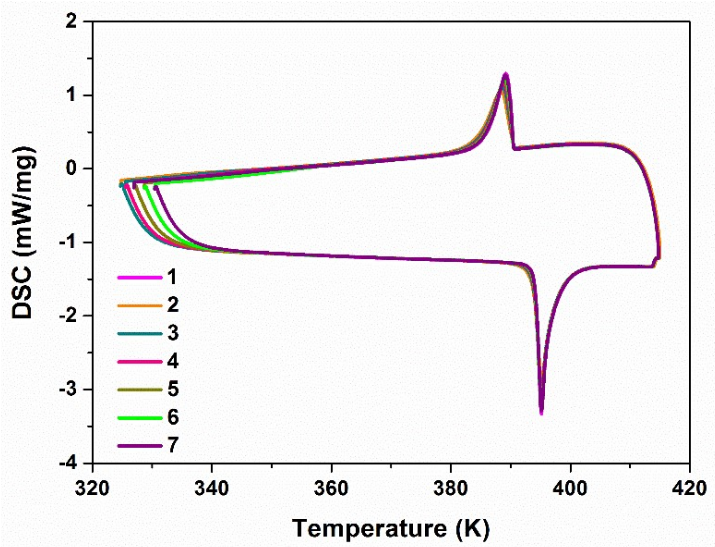

Fig. S8 DSC cycle test of compound **1** with the scan rate 15 K / min.

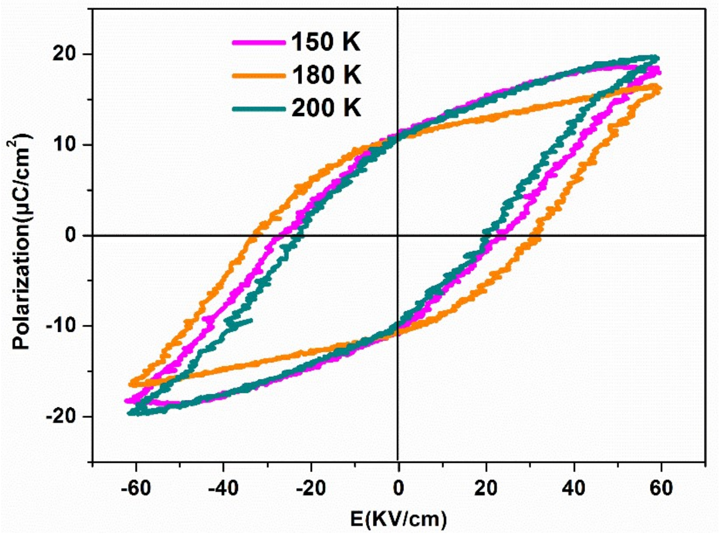

Fig. S9 Polarization hysteresis loops of compound **1** at low temperature.

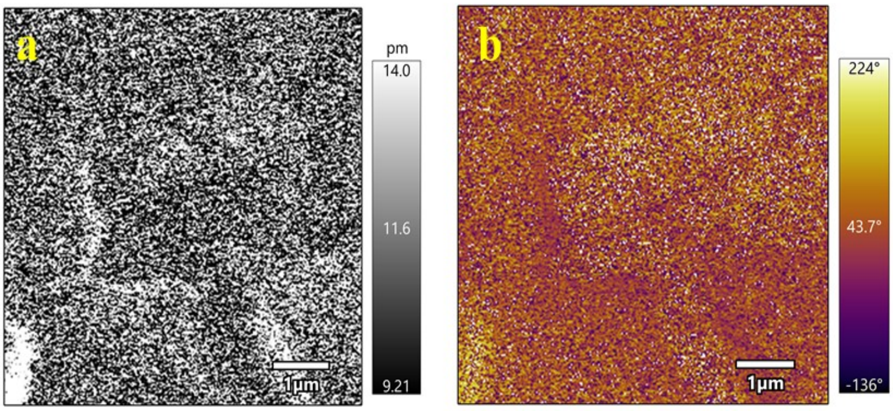

Fig. S10 The Vertical PFM amplitude (a) and phase (b) mapping.

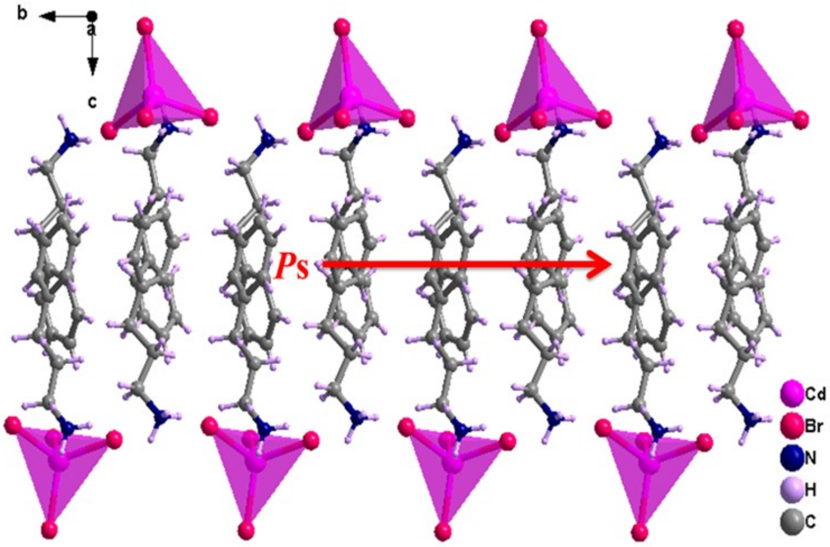

Fig. S11 Stacking diagram of compound **1**. The red arrow reveals the direction of P_s.

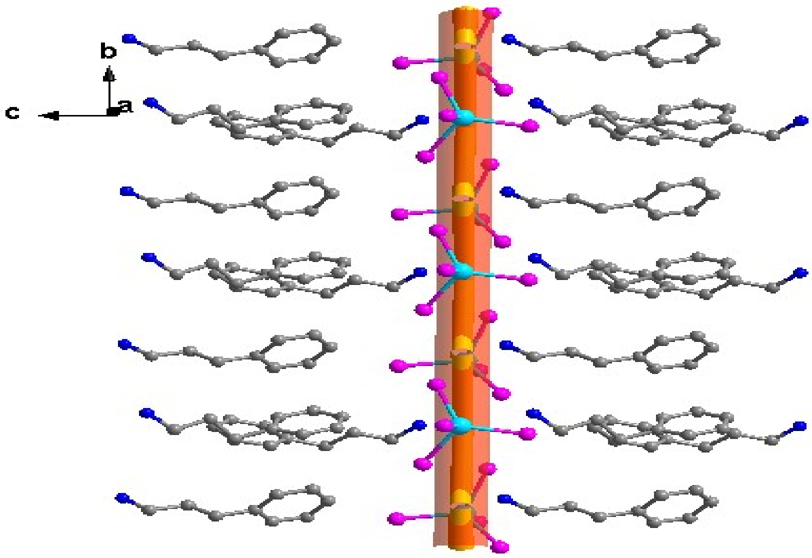

Fig. S12 $CdBr_4$ tetrahedrons are arranged around the spiral axis "S-shape".

Table.S1 Crystal data and refinement parameters for **1**

Compounds	**1**
Empirical formula formula	$C_{18}H_{28}Br_4CdN_2$
Temperature (K)	300 K
Crystal system	monoclinic
Space group	$P2_1$
a (Å)	11.1342(6)
b (Å)	7.8950(4)
c (Å)	13.9106(7)
α/°	90
β/°	96.953(2)
γ/°	90
V (Å^3)	1213.81(11)
Z	2
ρ calcg/cm^3	1.927
μ/mm^{-1}	7.488
F(000)	676.0
R_1, wR_2[I> 2σ(I)]	R_1 = 0.0499, wR_2 = 0.0807
R_1, wR_2(all data)	R_1 = 0.0770, wR_2 = 0.0898
Flack parameter	0.057(17)

Table S2. Selected bond lengths (Å) for Compound **1**

Compound	**1**	
Bond Lengths[Å]	Cd1-Br4	2.5788(13)
	Cd1-Br1	2.5998(13)
	Cd1-Br3	2.5961(13)
	Cd1-Br2	2.5599(14)
	N1-C1	1.466(13)

	N2-C10	1.446(15)
	C2-C1	1.491(13)
	C10-C11	1.492(15)

Table S3 Selected bond angles (°) for **1**

Compound	**1**	
Bond angles (°)	Br4-Cd1-Br1	104.81(5)
	Br4-Cd1-Br3	113.96(5)
	Br4-Cd1-Br1	106.48(4)
	Br2-Cd1-Br4	110.75(5)
	Br2-Cd1-Br1	107.25(5)
	Br2-Cd1-Br3	112.93(5)
	N1-C1-C2	112.5(9)
	N2-C10-C11	114.0(10)

Table S4 Hydrogen bonds parameters of **1**.

D–H···A	D–H	H···A	D···A	∠D-H···A
N1-H1A···Br1	0.89	2.74	3.495(9)	144
N1-H1B···Br4	0.89	2.69	3.476(9)	148
N1-H1C···Br2	0.89	2.74	3.556(9)	155
N2-H2C···Br1	0.89	2.61	3.424(10)	153
N1-H2D···Br3	0.89	2.73	3.502(10)	146
N1-H2E···Br3	0.89	2.57	3.374(10)	151

Calculation of ΔS and N

Compound 1:

In the heating cycle mode

$\Delta S\mathrm{H} = R\ln N1$

$$\Delta S\mathrm{H} = \int_{T_2}^{T_1} \frac{Q}{T} dT$$

$$\approx \frac{\Delta \mathrm{H}}{\mathrm{T}c}$$

$$= \frac{8.942 J^{-1} mol \times 704.46 g^{-1} mol}{395 K}$$

$=15.95 J\cdot mol^{-1}\cdot K^{-1}$

$$N_1 = \exp\left(\frac{\Delta S_H}{R}\right) = \exp\left(\frac{15.95 J \cdot mol^{-1} \cdot K^{-1}}{8.314 J \cdot mol^{-1} \cdot K^{-1}}\right)$$

$=6.75$

In the cooling cycle mode

ΔSC = $R\ln N2$

$$\Delta SC = \int_{T_2}^{T_1} \frac{Q}{T} dT$$

$$\approx \frac{\Delta H}{Tc}$$

$$= \frac{8.56 J^{-1} mol \times 704.46 g^{-1} mol}{389 K}$$

$=15.15 J\cdot mol^{-1}\cdot K^{-1}$

$$N_2 = \exp\left(\frac{\Delta S_c}{R}\right) = \exp\left(\frac{15.15 J \cdot mol^{-1} \cdot K^{-1}}{8.314 J \cdot mol^{-1} \cdot K^{-1}}\right)$$

$=6.17$
